# Incorporation of ZnO Nanoparticles into Soy Protein-Based Bioplastics to Improve Their Functional Properties

**DOI:** 10.3390/polym13040486

**Published:** 2021-02-04

**Authors:** Mercedes Jiménez-Rosado, Víctor Perez-Puyana, Pablo Sánchez-Cid, Antonio Guerrero, Alberto Romero

**Affiliations:** 1 Department of Chemical Engineering, Escuela Politécnica Superior, 41011 Sevilla, Spain; aguerrero@us.es; 2 Department of Chemical Engineering, Facultad de Química, 41012 Sevilla, Spain; vperez11@us.es (V.P.-P.); pabsanbue@alum.us.es (P.S.-C.); alromero@us.es (A.R.)

**Keywords:** bioplastics, nanoparticles, horticulture, soy protein-based

## Abstract

The union of nanoscience (nanofertilization) with controlled release bioplastic systems could be a key factor for the improvement of fertilization in horticulture, avoiding excessive contamination and reducing the price of the products found in the current market. In this context, the objective of this work was to incorporate ZnO nanoparticles in soy protein-based bioplastic processed using injection moulding. Thus, the concentration of ZnO nanoparticles (0 wt%, 1.0 wt%, 2.0 wt%, 4.5 wt%) and mould temperature (70 °C, 90 °C and 110 °C) were evaluated through a mechanical (flexural and tensile properties), morphological (microstructure and nanoparticle distribution) and functional (water uptake capacity, micronutrient release and biodegradability) characterization. The results indicate that these parameters play an important role in the final characteristics of the bioplastics, being able to modify them. Ultimately, this study increases the versatility and functionality of the use of bioplastics and nanofertilization in horticulture, helping to prevent the greatest environmental impact caused.

## 1. Introduction

The use of controlled release devices is increasing in the horticultural sector. In this way, products to release water [1], fertilizers [2] or pesticides [3] can be found in the market, improving their efficiency in crops. Generally, these devices are made using polymeric coatings that contain the substance to be released. This substance is released through the pores of the polymeric matrix due to diffusion between the matrix and the soil, thus reducing the leaching time of the substance and increasing the assimilation efficiency [4,5]. However, these devices have problems derived from the low biodegradability of the plastics used as matrices, which remain in the soil and are difficult to remove. This low biodegradability is due to the non-organic origin of these plastics, not following a natural decomposition process generated by bacteria. For this reason, bioplastic matrices are attracting attention in this application due to their biodegradability, which allows a controlled release of the fertilizer. In addition, this material does not release toxic substances and can be completely degraded without needing to be removed. In this way, these matrices can hold water and encapsulate fertilizers or pesticides. Subsequently, the substance can be controlled released through irrigation water or by biodegradation of the bioplastic matrix generated by different factors (i.e., change in pH or temperature, action of bacteria that degrade the polymeric matrix, irrigation) [6]. Thus, it can improve the assimilation efficiency and solve problems derived from the use of non-biodegradable plastics [7]. To this end, several studies have evaluated the use of bioplastic matrices in horticulture. For example, Olad et al. fabricated starch-based superabsorbent hydrogel from potato peel waste to retain and, subsequently, to control release of water to crops [8]. In this line, Michalik and Wandzinc studied chitosan-based hydrogels [9]. On the other hand, different nutrients have been incorporated into bioplastic matrices, such as sodium and NPK (primary nutrients: nitrogen, phosphorus and potassium) [10,11,12]. There are also examples of controlled release of pesticides, such as the work of Singh et al. [13], who used imidacloprid to release repellent odours for insects and microorganisms. All these studies use cheap raw materials (i.e., residues and byproducts of agri-food industry) whose costs are in the range 0.03–1.10 €/kg [14], which allows these products to be competitive with conventional plastics (with costs in the range of 0.57–1.59 €/kg) [7]. However, there are still no bioplastic matrices that allow different qualities to be combined, which would enable water, fertilizers and pesticides to be released together. Therefore, bioplastics have opened a new field in the horticultural sector, generating a new reorganization of the sector, increasing the efficiency of crops and reducing the amount of conventional plastics used. However, a lot of research is still needed before they can really compete with conventional plastics and even replace them. For instance, the manufacturing process should be optimized, together with the analysis of possible combinations between different applications in the same device.

In this sense, nanoscience provides a novel possibility to avoid these drawbacks. In this way, it allows the presence of pests and diseases to be detected from the beginning through nanoparticles that can activate a chemical or an electrical signal in the presence of contaminants like bacteria [15]. This signal can be recorded in sensors that allow farmers to act in time and save the crop [16]. In addition, it also allows the correct amount of fertilizers and pesticides that promote productivity to be applied, while ensuring environmental safety (avoiding the excessive use of fertilizers and pesticides), non-contamination of food and greater efficiency in the use of agricultural inputs, which reduces production costs [17]. In this context, new materials based on the use of metallic, polymeric and inorganic nanoparticles have been developed and characterized to increase crop productivity, as well as to improve the uptake and immobilization of nutrients by plants, an area called nanobiofertilization [18,19]. Moreover, these systems minimize the leaching of fertilizers into the subsoil and groundwater, preventing their contamination via nutrient excess. In addition, nanoparticles improve the absorption of nutrients by plants, mitigating eutrophication by reducing the transfer of nitrogen to underground aquifers [20]. Among them, zinc oxide (ZnO) nanoparticles are the most widely used, as they not only allow zinc to be supplied (which is an essential micronutrient for crop growth), but they also have a pesticidal nature, without contaminating the crop, or affecting its quality [21]. In this way, the use of ZnO nanoparticles instead of the salts conventionally used for fertilization generates an improvement in the assimilation efficiency of plants due to their smaller particle size, generating less leaching of nutrients to the subsoil (less contamination). In addition, their greater efficiency means that a lesser amount of product must be incorporated to supply micronutrient deficiencies and it is easier to obtain quality products without nutrient deficit problems. On the other hand, the ZnO nanoparticles generate other benefits such as their antibacterial activity that prevents pesticides from having to be used during cultivation. All this means that, although the commercial price of nanoparticles is higher than that of conventional salts, they are a promising alternative for their replacement [22,23]. These nanoparticles have already been used in other sectors, such as medicine, pharmacology and the food industry due to their antibacterial activity [24,25]. However, farmers are reluctant to use nanoparticles alone, due to their novelty, which does not allow their long-term impact on human health to be understood [26].

Although the incorporation of nanoparticles worsens the mechanical properties of bioplastics without incorporated nanoparticles, their incorporation allows these bioplastics to be used in horticulture for the controlled release of nutrients (in this case, zinc). In addition, it has great advantages over other bioplastics used for this purpose [27,28], to which conventionally used salts are incorporated, making bioplastics have even worse mechanical properties (making them difficult to handle during processing and distribution), further worsening their ability to absorb water and generating a faster release of the micronutrient. In addition, incorporating ZnO nanoparticles generates other additional benefits such as the antimicrobial activity that allows bioplastics can be used as a pesticide. All of this makes these bioplastics a great alternative to currently used devices on the market. For this reason, the objective of this work was to unite both concepts, bioplastics and nanoparticles, to create devices that allow the controlled release of water and fertilizers, while also acting as pesticides. The incorporation of nanoparticles into bioplastic matrices allows the amount of nanoparticles that need to be incorporated into the crop for its growth to be reduced, since they have greater efficiency than conventional fertilizers and would be incorporated in a controlled way in the crop, avoiding their possible leaching. All of this could reduce farmers’ reluctance to use them. Furthermore, this could grant bioplastic matrices more than one function, allowing them to compete with plastic devices. In this way, different concentrations of ZnO nanoparticles (0 wt%, 1.0 wt%, 2.0 wt% and 4.5 wt%) were incorporated into soy protein-based bioplastics processed via injection moulding. In addition, different mould temperatures (70 °C, 90 °C and 110 °C) were studied to evaluate the optimal parameters for their processing. To evaluate the different bioplastics, their mechanical, morphological and functional properties were measured.

## 2. Materials and Methods

### 2.1. Materials

The bioplastics were manufactured with soy protein isolate (SPI), which is a by-product obtained from soybean oil production (with 91 wt% protein), glycerol (Gly) and zinc oxide nanoparticles (ZnO). SPI and Gly were supplied by Protein Technologies International (Zwaanhofweg, Belgium) and Panreac Química Ltd. (Barcelona, Spain).

ZnO nanoparticles were synthesized from zinc chloride anhydride (ZnCl_2_) and sodium hydroxide (NaOH), both with 99% purity and provided by Panreac Química Ltd.

### 2.2. Synthesis of ZnO Nanoparticles

ZnO nanoparticles were synthesized using the colloidal precipitation method [29], using ZnCl_2_ as precursor and NaOH as reducing agent. For this, 20 mL of both ZnCl_2_ 0.2 M and NaOH 0.4 M solutions were magnetically mixed at 500 rpm for 2 h at 50 °C to promote reaction 1 (Equation (1)). Then, the zinc hydroxide (Zn(OH)_2_) was filtered and washed twice with 40 mL of distilled water in order to eliminate residues. Finally, this precipitate was dried in an oven (Memmert B216, Schwabach, Germany) at 100 °C for 8 h and calcined in a muffle furnace (Hobersal HD-230, Barcelona, Spain) at 500 °C for 4 h to promote the synthesis of ZnO nanoparticles according to reaction 2 (Equation (2)). All these conditions were selected to maximize the yield of the synthesis and minimize the particle size (Figure S1), for the nanoparticles to be within the range used in horticulture [22,23]. The assessment was made in a previous study (not yet published) evaluating the two factors that most alter yield and particle size: the concentration of ZnCl_2_ and ZnCl_2_:NaOH ratio used. Thus, the lowest concentrations of ZnCl_2_ are those that obtained the smallest particle sizes, having a higher yield when the ZnCl_2_:NaOH ratio used was 1:2.

ZnCl_2 (aq)_ + 2 NaOH _(aq)_ → Zn(OH)_2 (s)_ + 2 NaCl _(aq)_ (1)

Zn(OH)_2 (s)_ → ZnO _(s)_ + H_2_O _(g)_ (2)

### 2.3. Bioplastics Processing Method

Firstly, SPI and Gly (1:1) with different concentrations of ZnO nanoparticles (1.0 wt%, 2.0 wt% and 4.5 wt%) were homogenized in a Polylab QC (Themo Haake, Karlsruhe, Germany) for 30 min at 50 rpm under adiabatic conditions, beginning at 20 °C. During the mixing, the temperature and torque were monitored (Figure S2) to ensure that there was no plasticization in this stage (temperature < 37 °C (ΔT < 17%) and torque < 10 Nm) and that the blend was homogeneous and correctly mixed (values with a tendency to stabilize).

Later, the dough-like blends were subjected to injection moulding in a MiniJet Piston Moulding System II (Themo Haake, Karlsruhe, Germany). To this end, the blends were introduced in a cylinder at 40 °C, from which they were injected into a mould at a pressure of 600 bar for 20 s. The mould temperature was 70 °C, 90 °C or 110 °C. The bioplastic was kept in the mould for 20 s at 200 bar. The different mould temperatures were selected to evaluate the effect they have in the formation of the bioplastic with nanoparticles included. This temperature induces the formation of a gel-like network among protein chains, mainly through physical interactions and aggregation, which is reinforced by the presence of nanoparticles. Thus, a higher temperature of mould leads to an enhancement of the network.

Finally, bioplastics were subjected to a thermal treatment in a conventional oven at 50 °C for 24 h to strengthen the networks of bioplastics and, thus, maintain their physical integrity. This step has already been implemented in a previous research work for the same purpose [30].

### 2.4. Characterization of Bioplastics

#### 2.4.1. Mechanical Properties

A minimum mechanical requirement is necessary to guarantee the processability, transport and storage of bioplastics without damaging them. Mechanical properties (both flexural and tensile properties) were measured in rectangular bioplastics (60 × 10 × 1 mm^3^), using a dynamic-mechanical thermal analyser RSA3 (TA Instruments, New Castle, DE, USA) with a dual cantilever or rectangular geometry for flexural and tensile tests respectively.

##### Flexural Properties

Flexural measurements were performed, following a modification of ISO 178:2019 standard [31], in dynamic mode with a double bending geometry. For this, frequency sweep tests were carried out between 0.02 Hz and 20 Hz at a strain below the critical strain (0.05%) and at room temperature (22 ± 2 °C). Thus, the elastic (E’) and viscous (E’’) moduli caused by the application of a small amplitude oscillatory flexural strain were studied as a function of frequency. In addition, the loss tangent (tan δ = E’’/E’) was calculated in order to facilitate the evaluation of the behaviour of each bioplastic.

##### Tensile Properties

Tensile tests were carried out by applying a static axial force at a crosshead speed of 1 mm/min until bioplastic breakage at room temperature. In this way, maximum stress, strain at break and Young’s modulus were evaluated as differential parameters of tensile bioplastic behaviour. These tests were performed following a modification of ISO 570-2:1993 standard [32].

#### 2.4.2. Morphological Properties

Fundamentally, the mechanical and functional properties of bioplastics depend on their morphology, with their microstructure and the distribution of their components being of special interest.

##### Scanning Electron Microscopy (SEM)

Firstly, the bioplastics were subjected to SEM. For this, the samples were previously sputter-coated with palladium-gold and, subsequently, observed using a Zeiss EVO electron microscope (Pleasanton, CA, USA). Two different detectors were used, i.e., secondary electron and scattered electron detectors, to evaluate the microstructure and element distribution, respectively. In both cases, an acceleration voltage of 10 kV was used.

##### Energy Dispersive X-ray Analysis (EDXA)

In addition to scattered electron, an EDXA complement was coupled to the microscope to analyse the distribution of elemental concentration (Zeiss EVO electron microscope, Pleasanton, CA, USA). Thus, the different zones obtained in the scattered electron micrographs were evaluated to verify the composition of the bioplastic matrices.

#### 2.4.3. Functional Properties

##### Water Uptake Capacity (WUC)

One of the purposes of these bioplastics is to absorb and retain water without disintegrating in order to capture rain or irrigation water and supply it to the crop when needed. The ASTM D570-98 standard [33] was used to determine this property. In this way, the bioplastics were submerged in 300 mL of distilled water for 24 h, calculating their water uptake capacity and soluble matter loss as is indicated in previous works [28].

##### Nanoparticle Release

The study of nanoparticle release is essential for the evaluation of their performance as a controlled release fertilizer and pesticide. Different authors indicate that a quick way to measure this property is by controlling its release in water through conductivity measurements [34]. Thus, an EC-Metro (Crison BASIC 30, Barcelona, Spain) was used to measure the conductivity of the immersion water during the test. The maximum release time was obtained when the conductivity values remained stable for more than 30 min.

##### Biodegradability

The added value of these bioplastics is their degradation after their use without releasing toxic substances for crops, which eliminates the need for their removal. This quality was evaluated by burying the bioplastics in farmland and irrigating them daily with 20 mL of water (intensive irrigation simulation of 20 L water/m^2^). The samples were unearthed at different times, evaluating their disintegration using direct observation.

The used farmland was a special commercial substrate for orchards and fruit trees (Compo, Barcelona, Spain) which contains the ideal ratio of nutrient to the correct crop growth and does not contain microorganisms or pathogens that can alter the tests.

### 2.5. Statistical Analysis

All specimens were visually analysed prior to testing. In this way, those that presented defects were discarded from the study. The discarded specimens represented less than 10% of the specimens made.

Each analysis was carried out at least three times for each system and all the data were reported with their standard deviation statistically assessed using analysis of variance and Tukey’s post hoc test with 95% confidence level (*p* < 0.05) using the statistical package SPSS 18 (Excel, Microsoft, Redmond, WA, USA). The significant differences have been reported with different letters in the corresponding figures.

## 3. Results and Discussion

### 3.1. Mechanical Properties

#### 3.1.1. Flexural Properties

**Figure 1:**
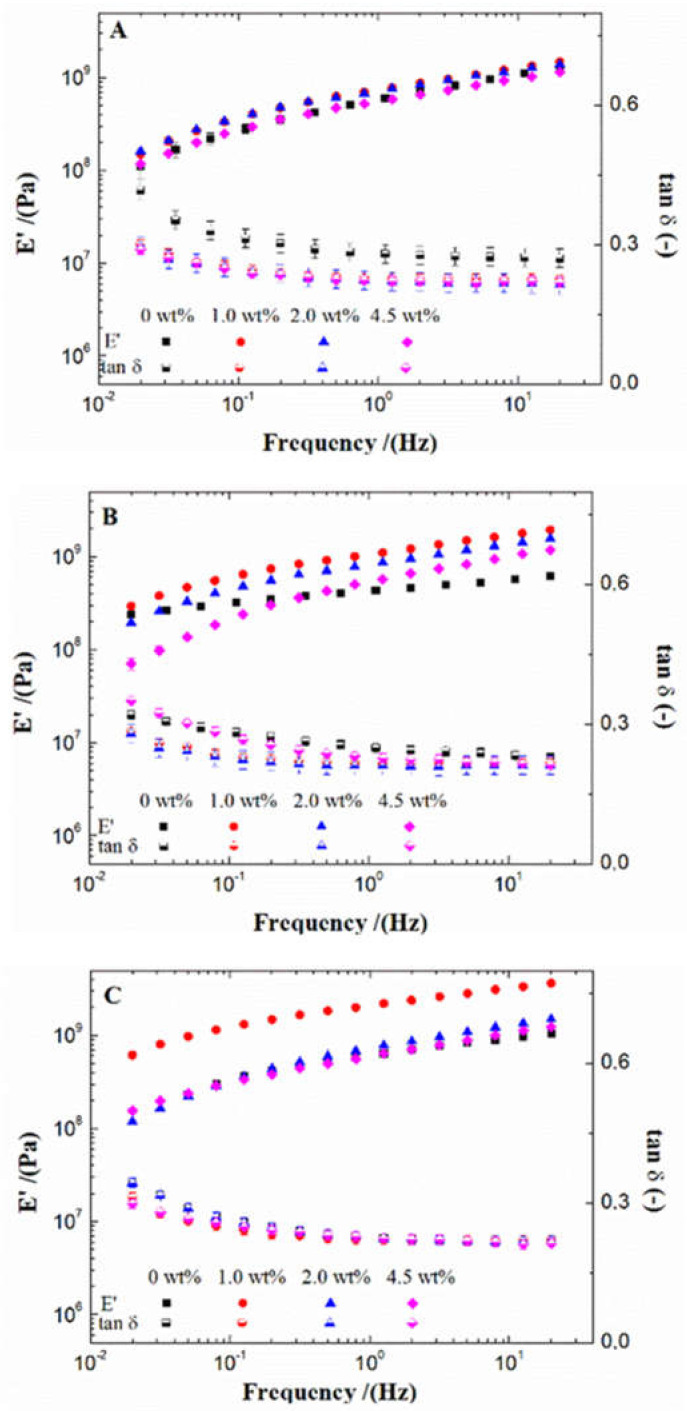
Flexural properties of bioplastics with different ZnO nanoparticle concentrations (0 wt%, 1.0 wt%, 2.0 wt% and 4.5 wt%) processed at different mould temperatures: 70 °C (**A**), 90 °C (**B**) and 110 °C (**C**). Elastic modulus (E’) and loss tangent (tan δ) profile in frequency interval.

Figure 1 shows the flexural properties of different bioplastics. All the systems have similar profiles in elastic modulus (E’) and loss tangent (tan δ), regardless of the micronutrient loading and mould temperature used. In this way, E’ increases with frequency at a rate that tends to become constant at high frequency, giving rise to a slope lower than 0.19. This behaviour may be due to an extension of the links that recover instantaneously, not leaving the linear range of deformations in the interval studied. This behaviour is similar to those obtained in other works with similar protein-based bioplastics, looking like a typical response from these materials [35,36].

The effect of the nanoparticle content on the elastic modulus depends on the mould temperature. Among the different mould temperatures used, 70 °C (Figure 1A) showed no significant differences between the different ZnO percentages used. However, this difference was more remarkable when the mould temperature used was 90 °C and 110 °C (Figure 1B and Figure 1C, respectively). In this sense, a low micronutrient load (1.0 wt% and 2.0 wt%) increased the E’ values, while higher loads (4.5 wt%) reduced it. In this case, at these temperatures, the incorporation of ZnO nanoparticles always induces an increase in the frequency dependence which is more apparent at 90 °C. The enhancement with micronutrient load, which is modulated by the mould temperature, could be attributed to the interaction between the nanoparticles and the bioplastic, as reported by other authors [37,38]. At low temperatures (70 °C), the nanoparticles, being in small concentrations, did not affect the bioplastics. However, the nanoparticles interacted with each other and with the biopolymeric chains when the temperature increased, improving the mechanical properties of the bioplastics [37]. Nevertheless, this improvement reached a limit, showing no increase in E’ values with 4.5 wt% nanoparticles. It can be kept in mind that a higher content of nanoparticles also involves a reduction in the protein content available that may impair the development of the protein network. Therefore, when the amount of filler material increased, it worsened the crosslinking between the biopolymer chains, thus limiting their mechanical properties. Similar behaviours have already been reported in previous studies, where filler materials improved the mechanical properties up to a certain concentration, reducing them at higher concentrations [39,40]. On the other hand, the bioplastics with 0 wt% and 1.0 wt% nanoparticles presented a slight increase in E’ values when higher mould temperatures are used, which is the common behaviour in these materials [41]. However, this increase is not observed at higher nanoparticle concentrations (2.0 wt% and 4.5 wt% ZnO).

Regarding tan δ, all systems presented similar values, between 0.2 and 0.35. This indicates that all bioplastics had a strong solid character that was enhanced either with the incorporation of nanoparticles or with the increase of temperature. This behaviour is characteristic in protein-based bioplastic, being found in other works. Thus, Yue et al. (2012) also found this behaviour in cottonseed protein [42], and the pea protein-based bioplastics processed by Perez et al. (2016) show this solid character [43]; Gómez-Heincke et al. (2017) obtained similar results with rice, potato and wheat gluten proteins [44].

#### 3.1.2. Tensile Properties

**Figure 2:**
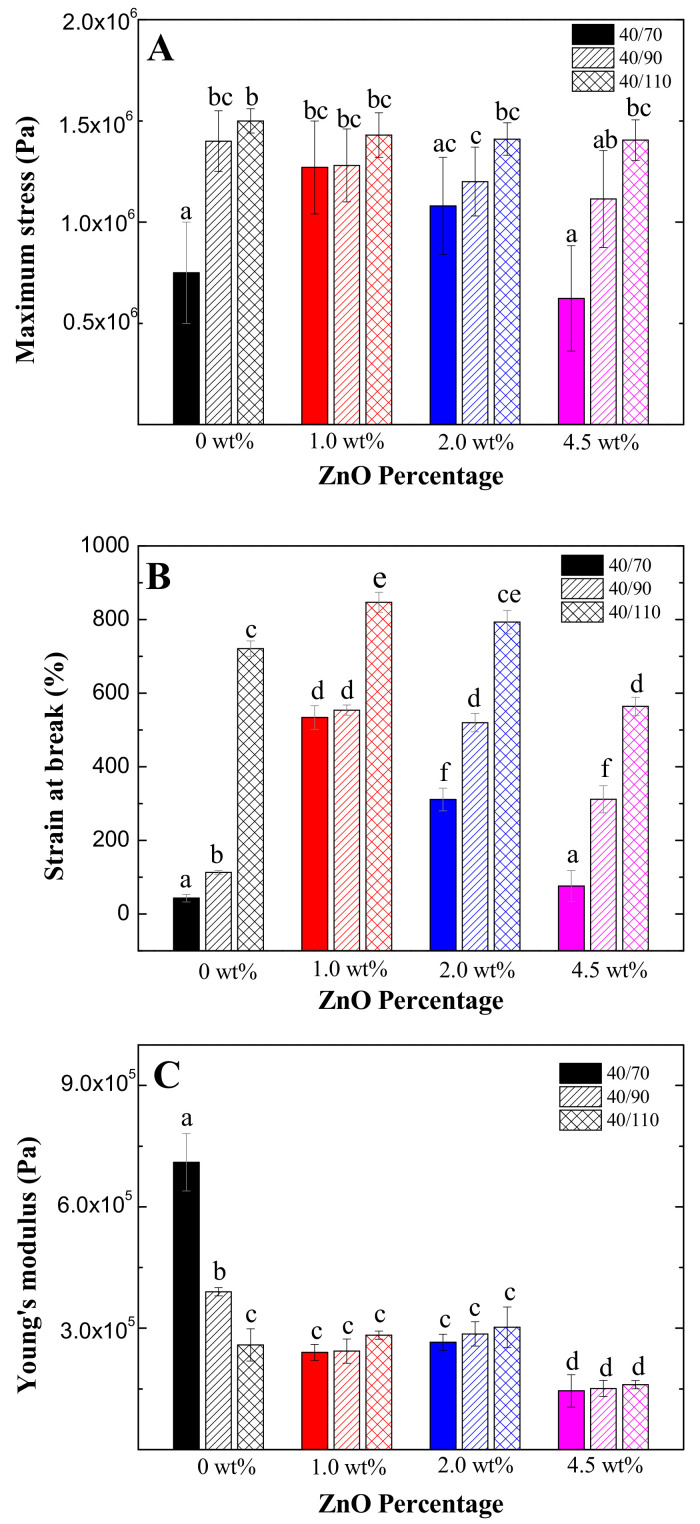
Tensile parameters of bioplastics with different ZnO nanoparticle concentrations (0 wt%, 1.0 wt%, 2.0 wt% and 4.5 wt%) processed at different mould temperatures (70 °C, 90 °C and 110 °C). (**A**): maximum stress. (**B**): strain at break. (**C**). Young’s modulus. Different letters in the bars mean that the values are significantly different (*p* < 0.05).

The tensile properties of bioplastics are shown in Figure 2. Firstly, the maximum stress (Figure 2A) increased when higher temperatures were applied, being more notable when the maximum stress started from lower values (0 wt% and 4.5 wt% nanoparticles). Furthermore, 1.0 wt% and 2.0 wt% nanoparticles increased the maximum stress at the same temperature, while 4.5 wt% nanoparticles decreased it. This behaviour is similar to those obtained with flexural properties, although for this parameter it is only significant at the lowest temperature. This evolution reaffirms the hypothesis of some detrimental effect on the development of the protein network caused by an excess of nanoparticles. The strain at break (Figure 2B) shows a similar behaviour as in maximum stress, although in this case, the effect is significant for all temperatures, and 1.0 wt% nanoparticles had significantly the highest values in this case.

On the other hand, Young’s modulus presented a different behaviour, which is opposite for the lowest mould temperature. Thus, an increase of mould temperature or content of nanoparticles caused a decrease in Young’s modulus, showing no significant differences once the minimum value was reached. This suggests that there is a minimum value of Young’s modulus that is not lost regardless of how the bioplastic is processed.

Finally, it is worth mentioning that all bioplastics show strong enough mechanical properties for the suitable transport, storage and distribution of the product, not altering its final functionality and facilitating its production. It is also interesting to point out that these ZnO-containing bioplastics show better mechanical properties than those formulated with zinc sulphate, which is an advantage in this sector [28].

### 3.2. Morphological Properties

**Figure 3:**
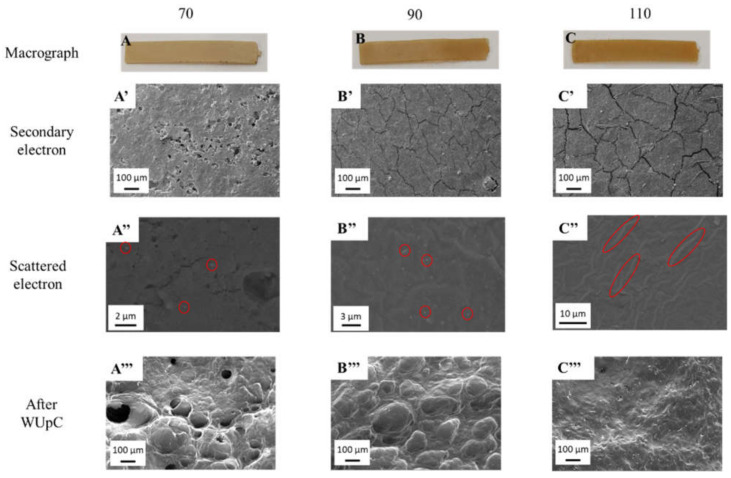
Macrographs (**A**–**C**) and micrographs of bioplastics with different ZnO nanoparticle concentrations (0 wt%, 1.0 wt%, 2.0 wt% and 4.5 wt%) processed at different mould temperatures (70 °C, 90 °C and 110 °C), using a secondary electron detector and a scattered electron detector before ((**A’**–**C’**) and (**A’’**–**C’’**), respectively) and after water uptake capacity (WUpC) tests (**A’’’**–**C’’’**).

The morphological properties of the bioplastics with 1.0% ZnO nanoparticles are shown in Figure 3 as the representative behaviour of all systems. Nevertheless, Figure S3 shows the morphology of the rest of the systems. Firstly, the macrographic images of the systems (Figure 3A–C) show that all the bioplastics are homogeneous, presenting a colour change with the increase of temperature. This colour change could be attributed to a higher degree of crosslinking generated by Maillard reactions, which colours the systems towards a tanner brown as the temperature increases. This change has already been observed in previous works [45,46]. However, a temperature change not only changes the macrographic appearance of the bioplastic, since differences in the microstructure are also noticed. In this way, structural differences can be seen in micrographic images obtained using a secondary electron detector (Figure 3A’–C’). The bioplastics processed at 70 °C were the only ones that presented porosity in their macrostructure, with cracks appearing in those processed at 90 °C and, above all, at 110 °C. This closure of the microstructure caused by temperature is due to the higher degree of crosslinking generated in these temperatures and has already been reported in previous works [35,41].

Furthermore, the distribution of ZnO nanoparticles in the bioplastics can be seen using a scattered electron detector (Figure 3A’’–C’’). All the systems present a homogeneous distribution of nanoparticles in the bioplastic, which appears as lighter areas within the dark matrix that makes up the bioplastic. These areas with different tonality were corroborated using EDXA as nanoparticles (white) and protein matrix (black) (Figure S4). As can be observed, the increase of mould temperature generates an effect of nanoparticles sintering, which join together, increasing their size [47], and even forming rings in the direction of injection. This behaviour of the ZnO nanoparticles with temperature could explain the structure observed by the secondary electron detector, since an increase in nanoparticle size makes it difficult to join the biopolymeric chains, causing the observed cracks to appear.

### 3.3. Functional Properties

#### 3.3.1. Water Uptake Capacity

**Figure 4:**
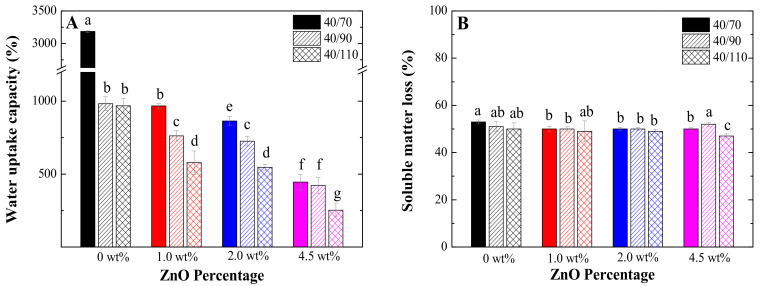
Water uptake capacity (**A**) and soluble matter loss (**B**) of the bioplastics with different ZnO nanoparticle concentrations (0 wt%, 1.0 wt%, 2.0 wt% and 4.5 wt%) processed at different mould temperatures (70 °C, 90 °C and 110 °C). Different letters (a,b, …, g) in the bars mean that the values are significantly different (*p* < 0.05).

Figure 4 shows the water uptake capacity (Figure 4A) and soluble matter loss (Figure 4B) of the different bioplastics. As can be seen, the increase in both temperature and ZnO nanoparticles percentage reduced the water uptake capacity of the bioplastic matrices, causing them to lose their superabsorbent quality. This behaviour could be due to the lower free volume and the greater crosslinking of the systems when mould temperature or nanoparticle percentage is increased, reducing the bioplastics’ space to interact with water, forming hydrogen bonds that retain it. This is corroborated using the SEM images after the water uptake capacity tests (Figure 3A’’’–C’’’), which show a decrease in pore size and depth. In addition, other research works have already reported this isolated behaviour when increasing the mould temperature [41] or the amount of additive incorporated [37,48] in similar bioplastics. Moreover, it is also worth mentioning that these bioplastics improve the water uptake capacity generated by systems studied for the same purpose, where microstructure salts were incorporated instead of nanoparticles, improving their functionality [27,28].

Regarding the soluble matter loss, there were no significant differences between the systems. This indicates that, even if the structure changes, the bioplastics always maintain their integrity by only releasing the incorporated plasticizer (glycerol) and part of the soluble protein.

#### 3.3.2. Nanoparticle Release

**Figure 5:**
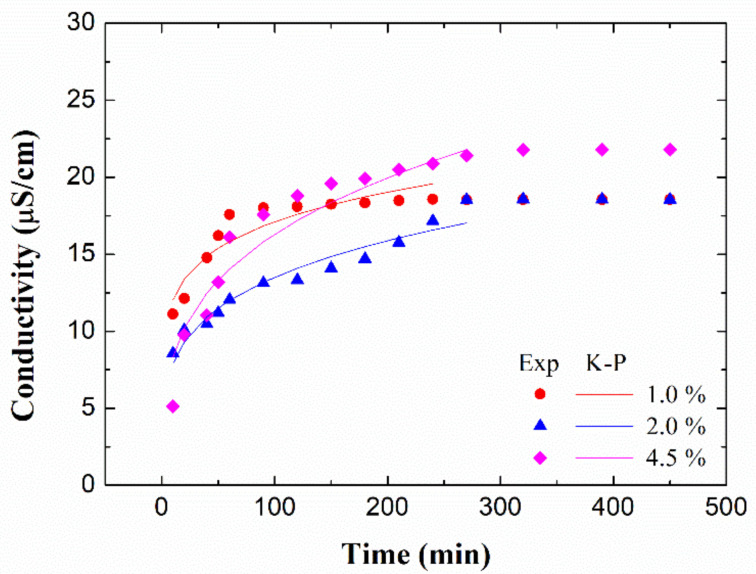
Accumulation of conductivity in the water release tests of bioplastics with different ZnO nanoparticle concentrations (1.0 wt%, 2.0 wt% and 4.5 wt%) processed at a mould temperature of 90 °C.

The profile of water release of nanoparticles is shown in Figure 5. However, only bioplastics processed with a mould temperature of 90 °C were shown as representative. As can be seen, all the systems present a quick release at short test times, probably due to the greater difference in concentrations between the system and the medium. This release stabilizes over time until reaching the maximum release time. Furthermore, all profiles adapt to the Korsmeyer-Peppas model with an *n* between 0.1 and 0.3, which indicates that several processes, such as diffusion, disintegration, etc., simultaneously occur during the release, none of them being predominant [49].

Regarding the maximum release time (Table 1), the higher the concentration of nanoparticles, the more prolonged the release over time. This indicates that all the incorporated nanoparticles are released in a controlled way. In addition, this maximum release time is higher than that found when microstructured salts are used instead of nanoparticles [27,50], which indicates that the release is better controlled on this occasion.

#### 3.3.3. Biodegradability

Finally, the degradation time of each bioplastic matrix is indicated in Table 1. As can be seen, higher mould temperatures lead to more durable bioplastics, probably due to the better mechanical properties observed with increasing temperature, as is reported in previous works [51]. However, the incorporation of nanoparticles in the bioplastics caused this degradation to be faster, except for the systems with 1.0 wt% nanoparticles. This behaviour could be due to the fact that, when the nanoparticles are released, there are more free holes where the bioplastic is more susceptible to degradation, thus accelerating this process. This behaviour has already been reported by Abdullah et al. (2020) [52]. Figure S5 shows the physical appearance of a bioplastic with 1.0 wt% nanoparticles processed at 110 °C. The appearance of bioplastics is similar in all cases, although the lightness of this decomposition changes. It should be noted that, in all cases, the bioplastics decompose into their primary elements (mainly nitrogen), serving as a supplementary fertilizer for the crop and not requiring its removal after use. Furthermore, the degradation time of bioplastics could be modified through the incorporation of nanoparticles and a change in mould temperature, making them very versatile, thus they could be used in all types of horticultural crops.

## 4. Conclusions

To sum up, soy protein-based bioplastics have shown their great capacity to hold ZnO nanoparticles and release them in a controlled way. In this sense, two novel lines of great interest in horticulture (bioplastics and nanobiofertilization) have been brought together, generating interesting synergies between them, and improving the devices investigated so far. Thus, a controlled release biodegradable device is achieved that presents functionality both to release water and fertilizers, as well as to be used as a long-lasting pesticide, having an enhanced efficiency in plants. In addition, these bioplastics have great versatility to change their characteristics by modifying their composition and processing parameters. In this way, they can be used in different crops, not being necessary to remove them after use. Nevertheless, this is only an initial study, requiring further research to evaluate the functionality of these bioplastics in real and large-scale crops.

## Figures and Tables

**Table 1:** Maximum release time of bioplastics with different ZnO nanoparticle concentrations (0 wt%, 1.0 wt%, 2.0 wt% and 4.5 wt%) processed at a mould temperature of 90 °C and degradation time of bioplastics with different ZnO nanoparticle concentrations (0 wt%, 1.0 wt%, 2.0 wt% and 4.5 wt%) processed at different mould temperatures (70 °C, 90 °C and 110 °C).

	Maximum Release Time (min)	Degradation Time (Days)		
		70 °C	90 °C	110 °C
0 wt%	-	40	60	70
1.0 wt%	240	40	60	70
2.0 wt%	270	20	40	50
4.5 wt%	390	10	20	30

## Data Availability

The data presented in this study are available on request from the corresponding author.

## Supplementary Materials

The following are available online at https://www.mdpi.com/2073-4360/13/4/486/s1, Figure S1: Yield (K_i_, A) and crystal size (B) of nanoparticles processed at different ZnCl_2_ concentrations (0.2, 0.5 and 1.0 M) and ZnCl_2_:NaOH ratios (0.5, 1.0 and 2.0); Figure S2: Temperature increment (A) and torque variation (B) of raw materials mixed at different nanoparticle concentrations (0, 1.0, 2.0 and 4.5 wt%); Figure S3: Macro and micrographs of bioplastics processed with 2.0 and 4.5 wt% ZnO nanoparticles at different mould temperatures (70, 90 and 110 °C); Figure S4: EDXA analyses of the different coloured zones in a bioplastic matrix with nanoparticles incorporated; Figure S5: Biodegradability photographs of bioplastics with 1.0 wt% ZnO nanoparticles processed at 110 °C.
