# Supplementary material for: Incorporation of ZnO Nanoparticles into Soy Protein-Based Bioplastics to Improve Their Functional Properties
Source: Polymers (Basel). 2021 Feb 4;13(4):486. doi: 10.3390/polym13040486 (PMC7913798; doi:10.3390/polym13040486)
Supplement: Supplementary file 1 [file polymers-13-00486-s001.pdf]

Supporting Materials

Incorporation of ZnO nanoparticles into soy protein-based bioplastics to improve their functional properties

Mercedes Jiménez Rosado [1,*], Víctor Perez-Puyana [2], Pablo Sánchez-Cid [2], Antonio Guerrero [1] and Alberto Romero [2]

[1] Department of Chemical Engineering, Escuela Politécnica Superior 41011 Sevilla, Spain, mjimenez42@us.es, aguerrero@us.es
[2] Department of Chemical Engineering, Facultad de Química 41012 Sevilla, Spain, vperez11@us.es, pabsanbue@alum.us.es, alromero@us.es
* Correspondence: e-mail: mjimenez42@us.es Tel.: +34 954 557 179

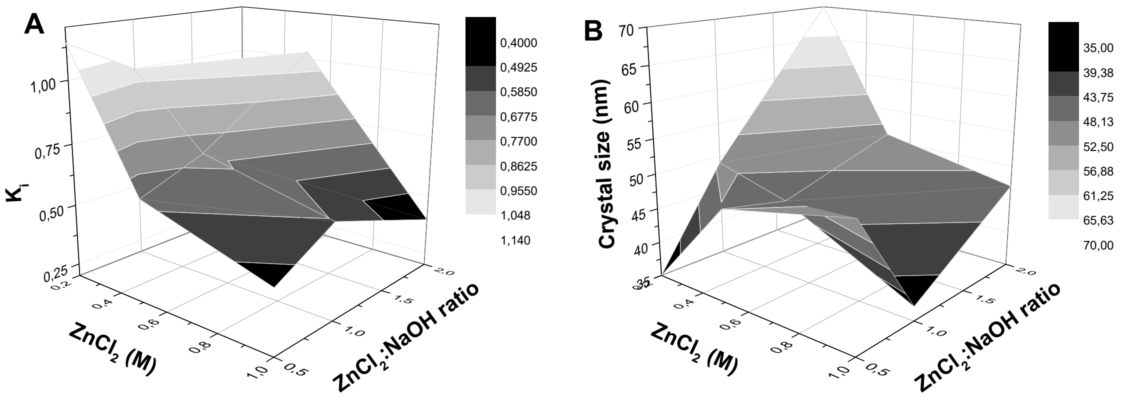

Figure S1. Yield (K_i, A) and crystal size (B) of nanoparticles processed at different $ZnCl_2$ concentrations (0.2, 0.5 and 1.0 M) and $ZnCl_2$:NaOH ratios (0.5, 1.0 and 2.0).

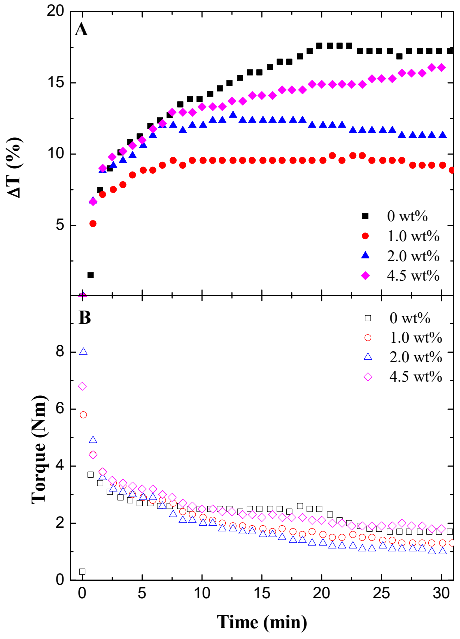

Figure S2. Temperature increment (A) and torque variation (B) of raw materials mixed at different nanoparticle concentrations (0, 1.0, 2.0 and 4.5 wt%).

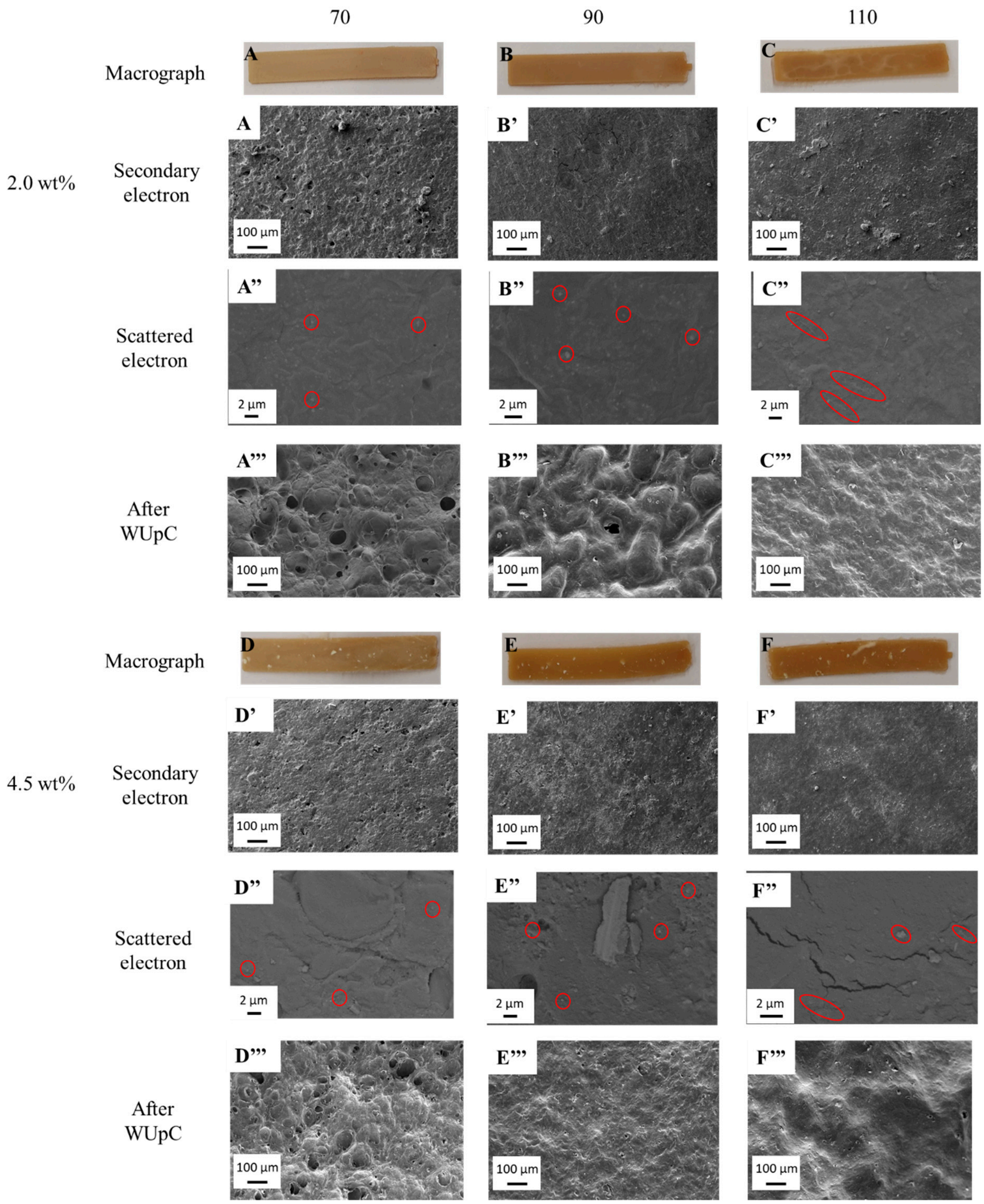

Figure S3. Macro and micrographs of bioplastics processed with 2.0 and 4.5 wt% of ZnO nanoparticles at different mould temperatures (70, 90 and 110 ^{0}C).

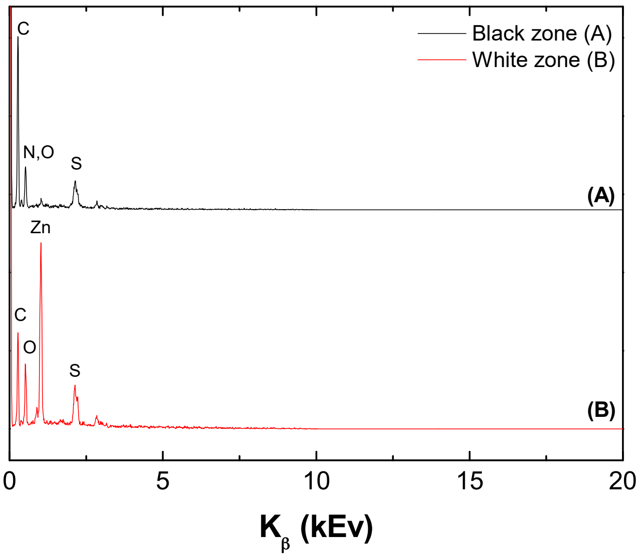

Figure S4. EDXA analyses of the different coloured zones (black and white zones) in a bioplastic matrix with nanoparticles incorporated.

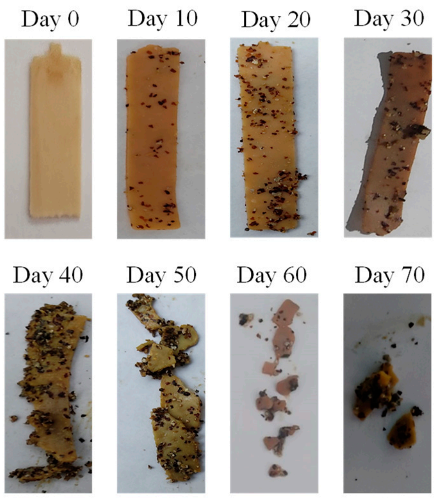

Figure S5. Biodegradability photographs of bioplastics with 1.0 wt% ZnO nanoparticles processed at 110 ⁰C.
